# Natural variation in *OsGASR7* regulates grain length in rice

**DOI:** 10.1111/pbi.13436

**Published:** 2020-07-06

**Authors:** Zhengbin Tang, Xiuying Gao, Xiangyun Zhan, Nengyan Fang, Ruqin Wang, Chengfang Zhan, Jiaqi Zhang, Guang Cai, Jinping Cheng, Yongmei Bao, Hongsheng Zhang, Ji Huang

**Affiliations:** ^1^ State Key Laboratory of Crop Genetics and Germplasm Enhancement College of Agriculture Nanjing Agricultural University Nanjing China; ^2^ Institute of Crop Sciences Fujian Academy of Agricultural Sciences Fuzhou China

**Keywords:** quantitative trait loci, grain length, genome‐wide association study, linkage mapping, gibberellic acid‐stimulated regulator, rice (*Oryza sativa*)

Identification of quantitative trait loci (QTLs) for grain length (GL) is important to rice breeding for increasing grain yield and appearance quality. Recent studies have identified a number of QTLs/genes as key grain length regulators by linkage mapping or genome‐wide association study (GWAS). These regulators are involved in G protein signalling, phytohormone signalling or transcriptional regulation, *etc* (Li *et al*., 2018). However, our current knowledge on GL is still fragmented in molecular mechanism and breeding utilization in rice. Here, we detected QTLs for GL by GWAS using 210 rice accessions from rice diversity panel 1 (RDP1) (McCouch *et al*., 2016) and linkage mapping using a recombinant inbred line (RIL) population with 116 lines derived from *geng*/*japonica* rice Suyunuo (long grain) and Bodao (short grain) (Figure 1a). Interestingly, one co‐localized locus was identified and *LOC_Os06g15620* encoding a gibberellic acid‐stimulated regulator (GASR) protein included in this region was confirmed to control GL.

**Figure 1:**
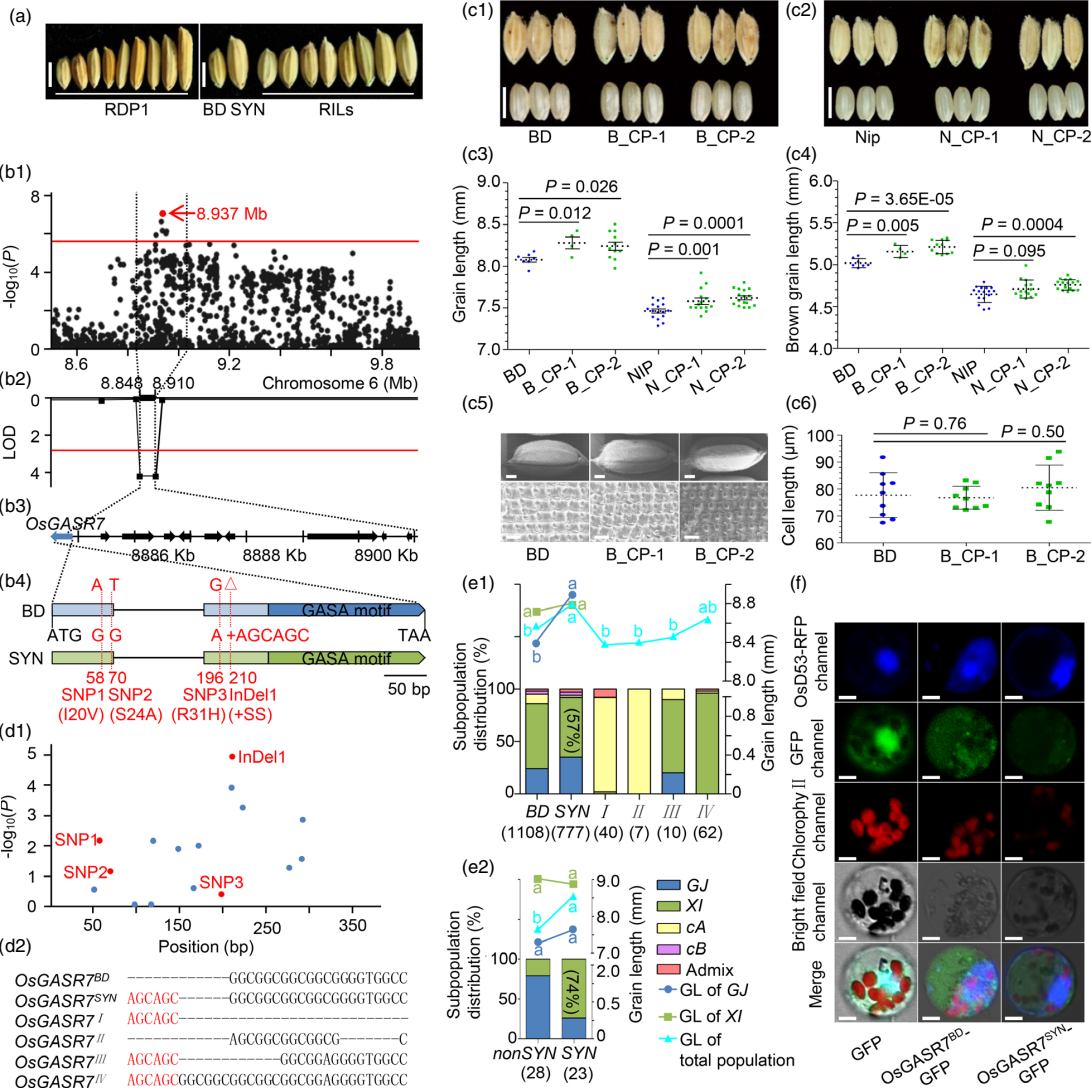
Cloning and variation analysis of *OsGASR7* for grain length in rice. (a) Grain morphology of Bodao (BD), Suyunuo (SYN) and some representative lines/accessions from RILs/RDP1. Scale bar, 5 mm. (b) Identification and positional cloning of *OsGASR7* combining GWAS and linkage mapping. (b1) The genome‐wide association signals for grain length are shown in the region at about 8.5–10.0 Mb on chromosome 6 (*x*‐axis). Negative log10‐transformed *P*‐values from the EMMAX algorithm are plotted on the *y*‐axis. The position of the peak SNP is indicated by the red dot. The horizontal red line indicates the threshold (−log10(*P*) = 5.6). (b2) The QTL for grain length is shown in the region at 8.848–8.91 Mb on chromosome 6 (*x*‐axis). LOD values from ICIM‐ADD algorithm are plotted on the *y*‐axis. The horizontal red line is the LOD threshold (2.82). (b3) The physical position of the predicted ORFs (filled arrows). (b4) Non‐synonymous variants of *OsGASR7* between Bodao and Suyunuo. Black lines represent introns, and colour bars represent exons. (c) *OsGASR7* controls grain length. (c1, c2) Grain morphology of wild‐type Bodao (BD), Nipponbare (Nip) and transgenic complemental rice BD‐p*OsGASR7^SYN^*::*OsGASR7^SYN^* (B_CP), Nip‐p*OsGASR7^SYN^*::*OsGASR7^SYN^* (N_CP). Scale bar, 5 mm. (c3, c4) Statistical analysis of (brown) grain length of wild‐type plants and transgenic plants. The dashed lines and error lines represent means ± *SD* (*n* ≥ 4 plants, shown by dots). (c5) SEM observation of mature seeds. Scale bar, 1 mm for seed and 100 μm for lemma. (c6) Statistical analysis of cell length of the glume outer surfaces. Data are presented as means ± *SD* (*n* = 9). The *P*‐values are calculated by Student's *t*‐test. (d) InDel1 is significantly associated with grain length. (d1) The *x*‐axis indicates position of each variation in the ORF of *OsGASR7* and the *y*‐axis is negative log10‐transformed *P*‐values from GLM algorithm. (d2) Six major genotypes of *OsGASR7*. (e) Genotype analysis of *OsGASR7* for subpopulation distribution and grain length in 3K accessions (e1) and cultivars (e2). *GJ*: *geng/japonica*; *XI*: *xian*/*indica*; *cA*: *centrum*‐*Aus*; *cB*: *centrum*‐*Basmati*; *BD*: *OsGASR7^BD^*; *SYN*: *OsGASR7^SYN^*; I: *OsGASR7^I^*;II: *OsGASR7^II^*; III: *OsGASR7^III^*; Ⅳ: *OsGASR7^Ⅳ^*; *nonSYN*: cultivars without *OsGASR7^SYN^*; number in brackets: number of statistical samples. Significance analysis is performed by Student's *t*‐test. (f) The subcellular localization of OsGASR7^BD^‐GFP and OsGASR7^SYN^‐GFP. Scale bar, 5 μm.

A GWAS performed for GL using the efficient mixed‐model association eXpedited (EMMAX) algorithm approach identified an associated locus over the threshold on chromosome 6 (−log_10_(1/401 085) ≈ 5.6) (Figure 1b1). This locus was further narrowed down to a 62‐Kb region by linkage mapping (Figure 1b2). According to the Rice Genome Annotation Project (http://rice.plantbiology.msu.edu/), 10 annotated genes were located within the above‐described locus (Figure 1b3). Among them, a candidate gene *OsGASR7* was also previously identified in a GWAS analysis for GL (Huang *et al*., 2012) and was found to be responsive to gibberellins and brassinosteroids in rice (Wang *et al*., 2009). Moreover, the wheat orthologous counterpart of *OsGASR7*, *TaGASR7*‐*A1*, was found to affect GL in common wheat under multiple cultivation conditions (Dong *et al*., 2014), and its CRISPR/Cas9‐induced *aabbdd* mutant significantly elevated thousand kernel weight (Zhang *et al*., 2016). Recently, OsGASR7/GW6 was found to regulate grain width and weight (Shi *et al*., 2020). Thus, *OsGASR7* is likely a candidate for controlling GL. *OsGASR7* contains a 437‐bp open reading frame (ORF) with two exons and one intron, and the sequence comparison of exons between Bodao and Suyunuo revealed three SNPs and one InDel (Suyunuo to Bodao: SNP1, 58G → A; SNP2, 70G → T; SNP3, 196A → G; InDel1, +AGCAGC between 209C and 210G). These variations lead to three amino acid substitutions and additional two serines (Figure 1b4).

To confirm the effect of *OsGASR7* on grain length, the functional complementation test of *OsGASR7* was performed. We introduced the entire genomic region of *OsGASR7^SYN^* from Suyunuo (SYN) into Bodao (BD) and Nipponbare (Nip) which carries *OsGASR7^BD^*, respectively, and generated two types of transgenic complemental rice, BD‐p*OsGASR7^SYN^*::*OsGASR7^SYN^* (B_CP) and Nip‐p*OsGASR7^SYN^*::*OsGASR7^SYN^* (N_CP). As expected, both BD‐p*OsGASR7^SYN^*::*OsGASR7^SYN^* and Nip‐p*OsGASR7^SYN^*::*OsGASR7^SYN^* exhibited increases in GL, confirming the role of *OsGASR7* in controlling GL (Figure 1c1–c4). As grain elongation is related to cell division and/or cell expansion, the outer lemma surfaces of mature seeds were examined by scanning electron microscopy (SEM). The results showed that there was no significant difference in cell length between Bodao and BD‐p*OsGASR7^SYN^*::*OsGASR7^SYN^* (Figure 1c5, c6), indicating the involvement of *OsGASR7* in cell division during spikelet hull development to control GL.

In order to further explore the variations in *OsGASR7* in controlling GL, we conducted *OsGASR7*‐based candidate gene association analysis using 2004 accessions from RFGB (Wang *et al*., 2019) by general linear model (GLM) algorithm. The association analysis showed that the InDel1 of 6‐bp insertion (AGCAGC) was the most significantly associated with GL (Figure 1d1). Interestingly, InDel1 locates in the variable region containing a polyglycine tract of OsGASR7, which is specific in cereal species including common wheat (Dong *et al*., 2014). It indicates that InDel1 is critical for grain length regulation, and we thus divided *OsGASR7* into six major genotypes based on the variable region where InDel1 locates (*OsGASR7^BD^*, *OsGASR7^SYN^*, *OsGASR7* ^*I*^, *OsGASR7* ^*II*^, *OsGASR7* ^*III*^ and *OsGASR7^Ⅳ^*) (Figure 1d2).

In the 2004 rice accessions, 55.3%, 38.8%, 2.0%, 0.3%, 0.5% and 3.1% of them carry *OsGASR7^BD^*, *OsGASR7^SYN^*, *OsGASR7*I, *OsGASR7*II, *OsGASR7*III and *OsGASR7^Ⅳ^*, respectively, and *OsGASR7^SYN^* has the strongest effect on GL no matter in *xian*/*indica* (*XI*), *geng*/*japonica* (*GJ*) or total population (Figure 1e1). Especially in *GJ*, the grains of rice with *OsGASR7^SYN^* are significantly longer than that without *OsGASR7^SYN^* (Figure 1e1), and this is consistent with the results of complementation test. Although containing the InDel1, rice accessions with *OsGASR7^I^* and *OsGASR7^III^* exhibit short grains. However, these rare alleles also show variations in flanking sequence of InDel1 compared to *OsGASR7^SYN^*, indicating that the flanking sequence or the position of InDel1 may also affect the grain length regulation. To find out whether *OsGASR7^SYN^* has been utilized in breeding, we analysed *OsGASR7* sequences of 42 cultivars. Compared with the distribution of *OsGASR7^SYN^* in *XI* of 2004 accessions, that in *XI* cultivars has increased (57% versus 74%) (Figure 1e). What is more, the grains of cultivars with *OsGASR7^SYN^* are still longer than those without *OsGASR7^SYN^* (*non‐OsGASR7^SYN^*) (Figure 1e2). These results show that *OsGASR7^SYN^* has been likely selected in many *XI* cultivars.

At last, we examined the subcellular localization of OsGASR7^SYN^ and OsGASR7^BD^ protein carrying green fluorescent protein (GFP) in rice protoplast expressing a nuclear marker OsD53‐mCherry. Both fluorescence of OsGASR7^SYN^‐GFP and OsGASR7^BD^‐GFP were mainly observed in cytoplasm (Figure 1f). Through *in silico* gene expression analysis, it was found that *OsGASR7* was regulated by brassinolide treatment and accumulated in panicles, suggesting that OsGASR7 may involve brassinosteroid (BR) pathway to regulate grain length in rice.

Functions of some GASR genes have been studied in rice. *OsGASR1* controls seedling growth and amylase production (Lee *et al*., 2017), and *OsGASR9* plays a positive role in the response to GA and grain development (Li *et al*., 2019). In this work, we confirmed that a GASR protein OsGASR7 is responsible for grain length regulation in rice by GWAS, linkage analysis and transgenic complemental study. More importantly, a natural variation of 6‐bp insertion (AGCAGC) in *OsGASR7* was found to be significantly associated with GL and could be potentially used as a molecular marker for rice breeding. The allele variations in different rice cultivars may lead to the functional diversity of *OsGASR7* observed in the studies of *GW6* (Shi *et al*., 2020) and this work. Further studies will be conducted to clarify how OsGASR7 modulates grain length and the role of additions of two serines in OsGASR7 function and regulation.

## Conflicts of interest

The authors declare no conflict of interest.

## Author contributions

J.H. and H.Z. contributed to the original concept of the project. Z.T., N.F., R.W., C.Z. and J.Z. performed the experiments and analysed the data. X.Z. and G.C. were responsible for material planting in field. J.C., X.G. and Y.B. participated in the design and coordination of the study. Z.T. and J.H. wrote the manuscript. All authors read and approved the final version of the manuscript.
